# Supplementary figures and images for: Prevalence and genome features of lake sinai virus isolated from Apis mellifera in the Republic of Korea
Source: PLoS One. 2024 Mar 19;19(3):e0299558. doi: 10.1371/journal.pone.0299558 (PMC10950237; doi:10.1371/journal.pone.0299558)

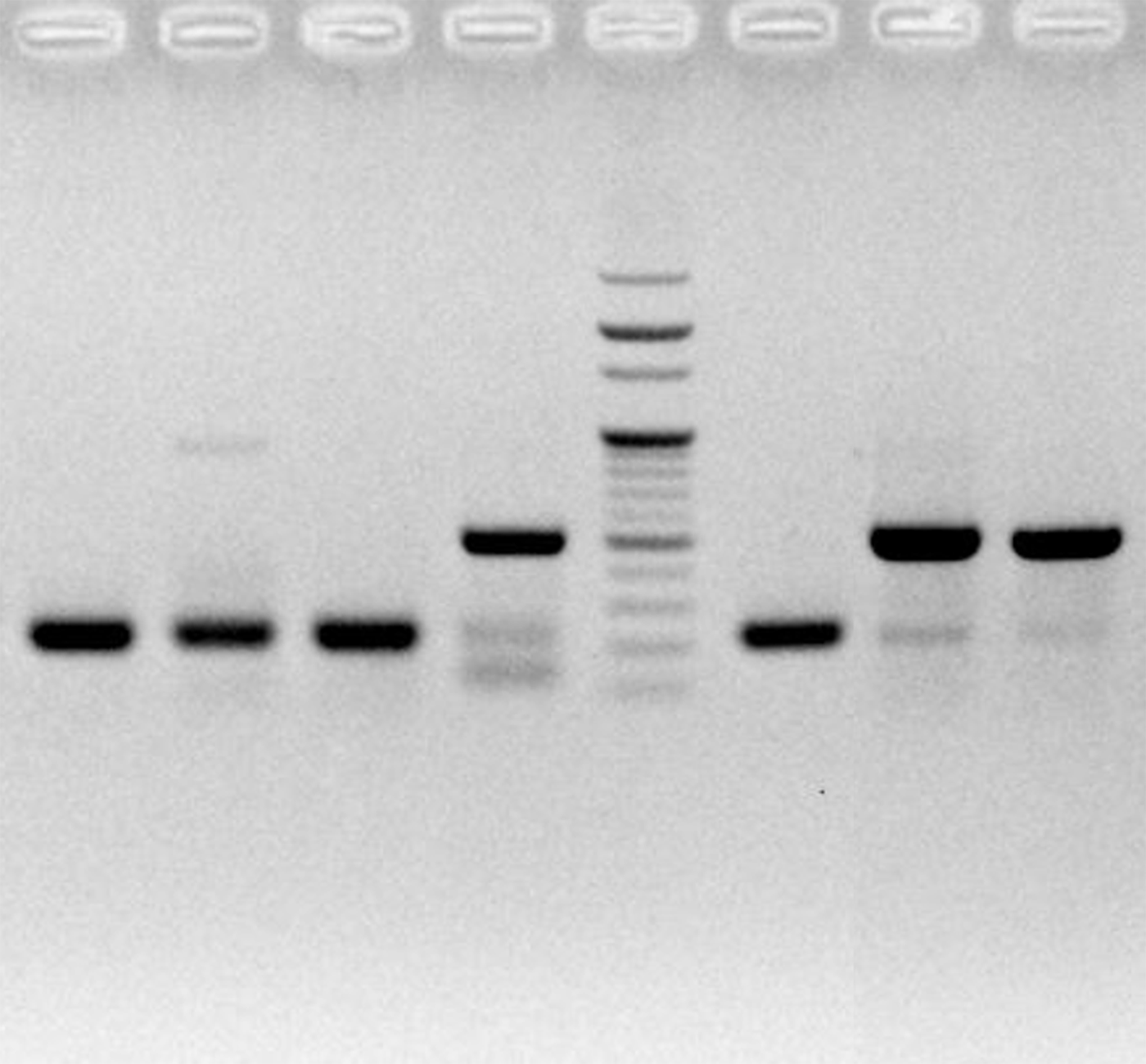

Supplement: S1 Fig — The positions of the forward and reverse primers inside the RdRp-encoding genes are marked. Sequences of each LSV genotype with NCBI accession numbers are shown. (ZIP) [file pone.0299558.s001.zip › S1c_Fig.tif]

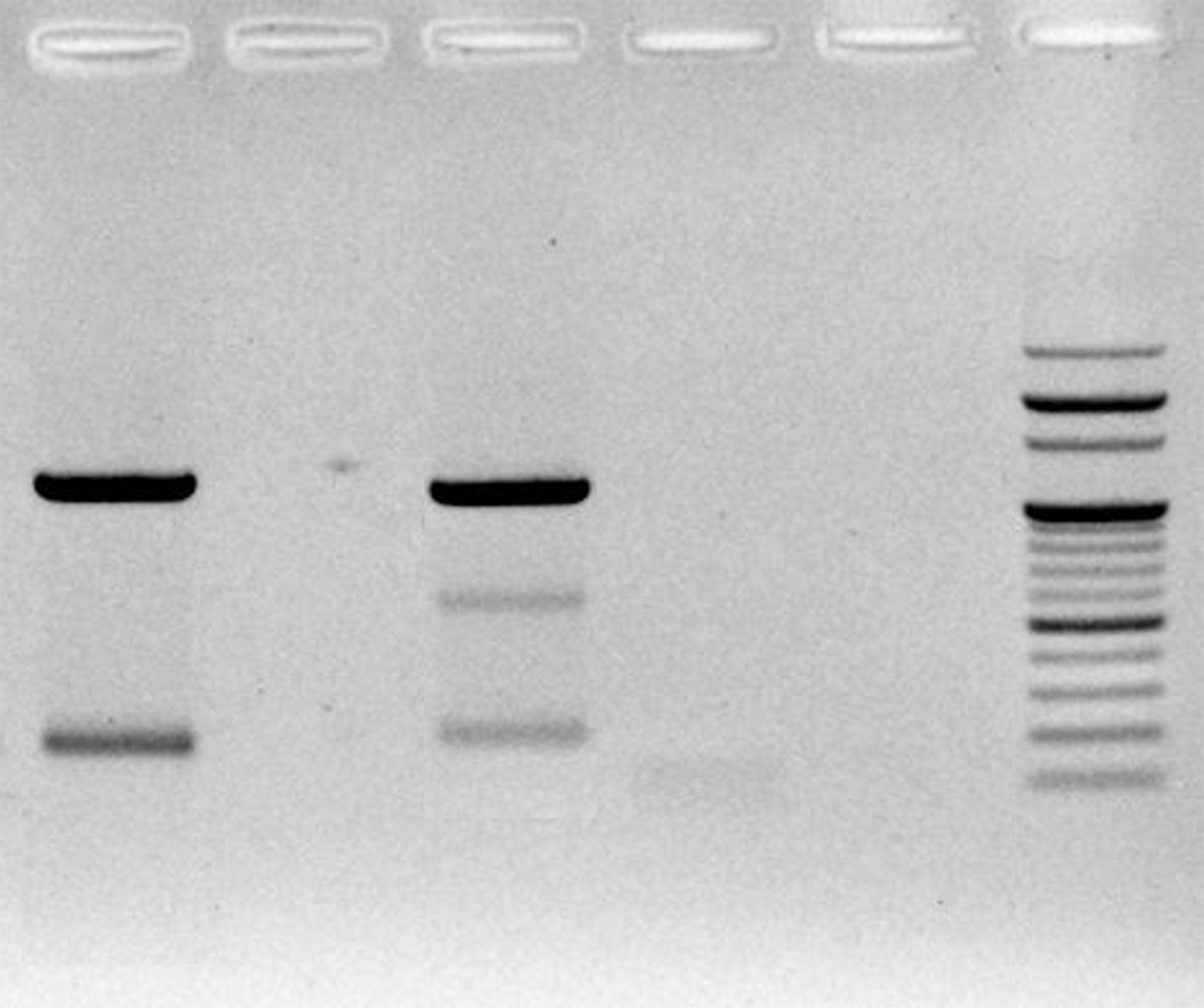

Supplement: S1 Fig — The positions of the forward and reverse primers inside the RdRp-encoding genes are marked. Sequences of each LSV genotype with NCBI accession numbers are shown. (ZIP) [file pone.0299558.s001.zip › S1d_Fig.tif]

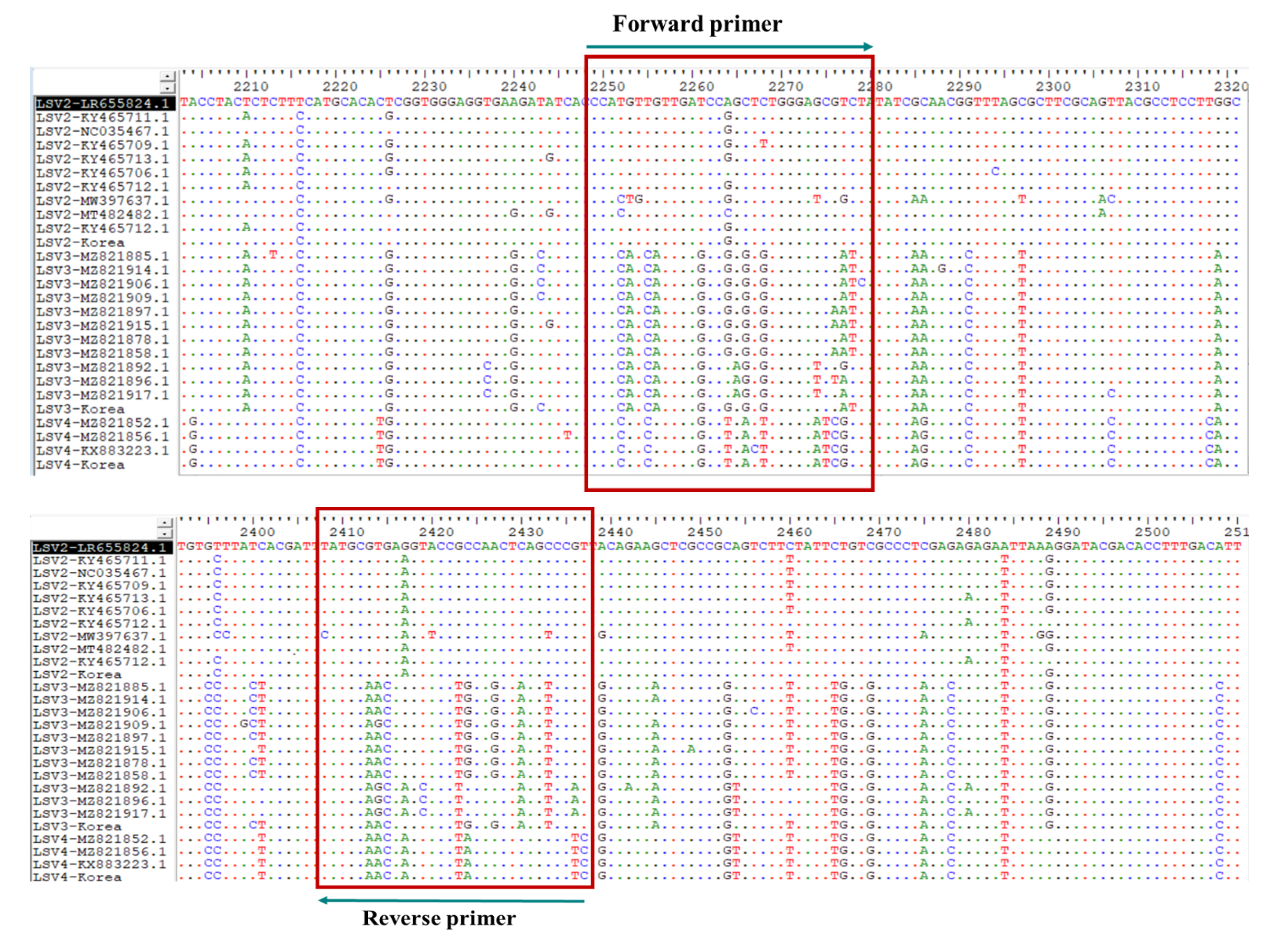

Supplement: S1 Fig — The positions of the forward and reverse primers inside the RdRp-encoding genes are marked. Sequences of each LSV genotype with NCBI accession numbers are shown. (ZIP) [file pone.0299558.s001.zip › S1a_Fig.tif]

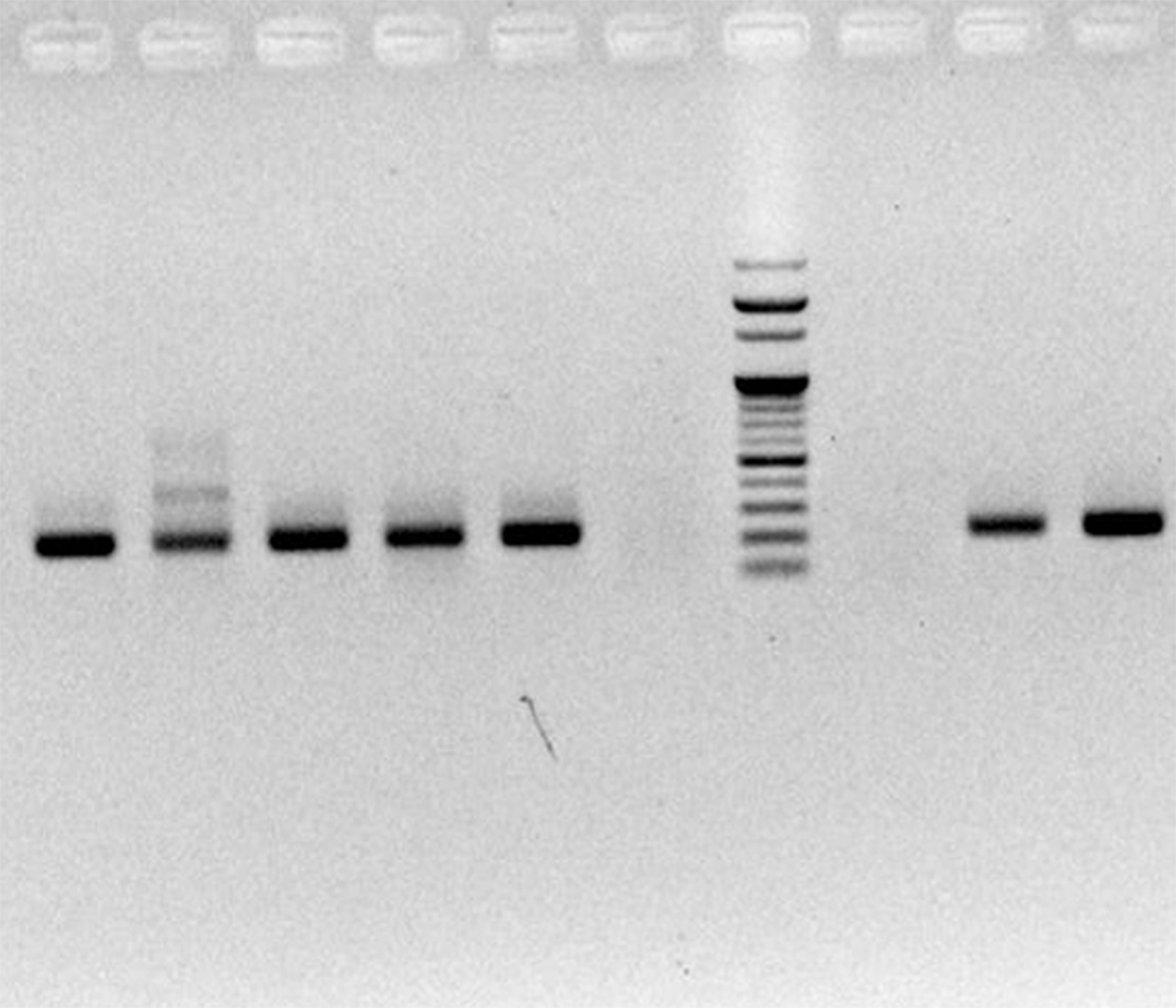

Supplement: S1 Fig — The positions of the forward and reverse primers inside the RdRp-encoding genes are marked. Sequences of each LSV genotype with NCBI accession numbers are shown. (ZIP) [file pone.0299558.s001.zip › S1b_Fig.tif]

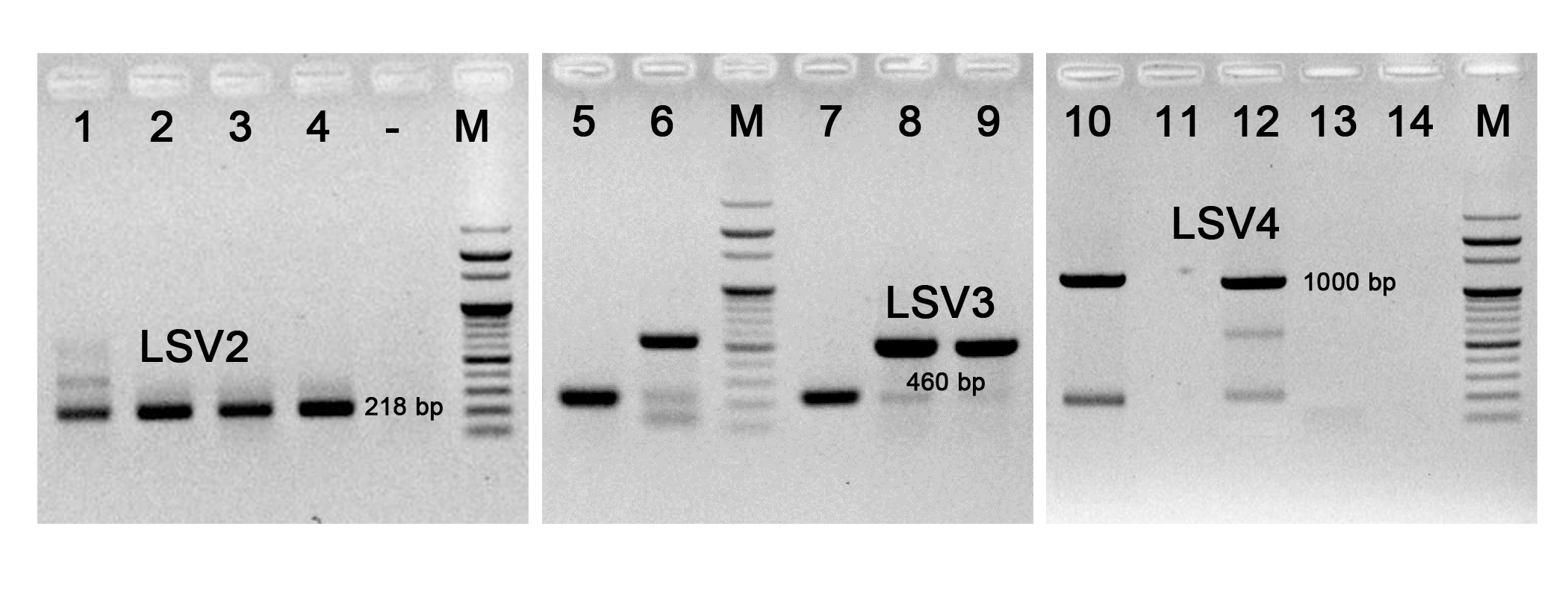

Supplement: S2 Fig — The PCR products were amplified using an LSV2-specific primer pair. Lanes 1–4, 5, and 7 show the PCR products of the LSV amplification of LSV2 (218 bp); lanes 6, 8, and 9 show the bands for LSV3 (460 bp); lanes 10 and 12 show bands for LSV4 (1,000 bp); lane “-”, Negative control; lane M, 100 bp DNA marker ladder (Enzynomics, Daejeon, ROK). (TIF) [file pone.0299558.s002.tif]

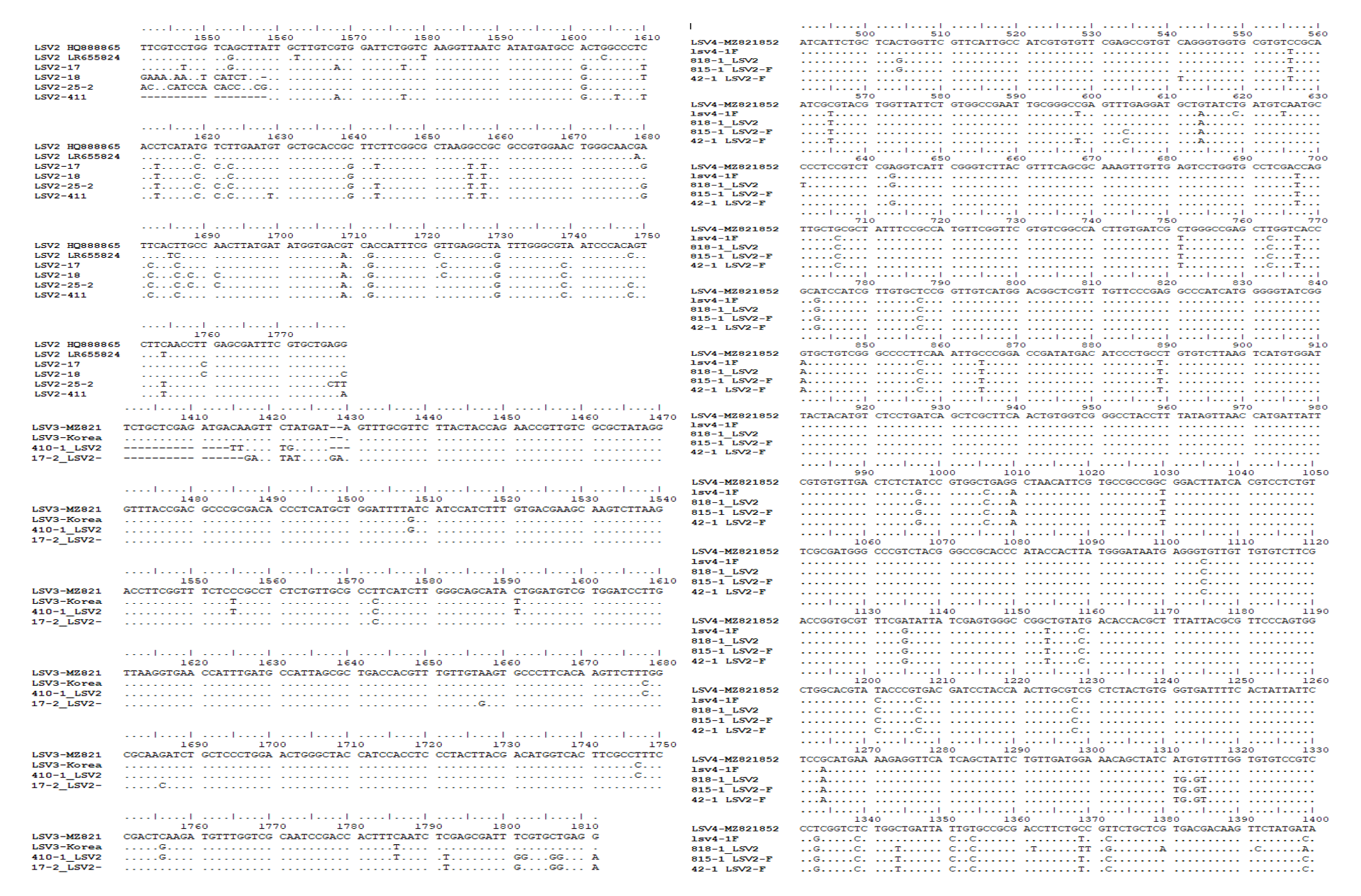

Supplement: S3 Fig — The sequences of LSV2, LSV3, and LSV4 isolated from honeybee samples were sequenced using forward and reverse primers of LSV2. The reference sequence of each LSV genotype with its NCBI accession number is shown. (TIF) [file pone.0299558.s003.tif]
