# Supplementary material for: Prevalence and genome features of lake sinai virus isolated from Apis mellifera in the Republic of Korea
Source: PLoS One. 2024 Mar 19;19(3):e0299558. doi: 10.1371/journal.pone.0299558 (PMC10950237; doi:10.1371/journal.pone.0299558)
Supplement: S2 Table — (DOCX) [file pone.0299558.s005.docx]

**S2 Table. Primers used for detecting LSV2, LSV3, and LSV4.**

| **No.** | **Primer Name** | **Sequences (5′–3′)** | **Position** | **Amplicon size (bp)** | **Reference** |
| --- | --- | --- | --- | --- | --- |
| 1 | LSV2-RT-For | CCATGTTGTTGATCCGGCTCTGGGAGCGTC | 2244-2432 | 188 | This study |
|  | LSV2-RT-Rev | ACGGGCTGAGTTGGCGGTACTTCACGCATA |  |  |  |
| 2 | LSV3-RT-For | CCACATCATTGAGCCGGGTGTGGGAGCGAT | 2238-2426 | 188 |  |
|  | LSV3-RT-Rev | ACGGACTTAGCTGCAGGTACCTGTTGCATA |  |  |  |
| 3 | LSV4-RT-For | CCACGTCGTTGAGCCTGATTTGGGAATCGC | 2239-2427 | 188 |  |
|  | LSV4-RT-Rev | GAGGGCTGAGTTGTAGGTACTTGTTGCATA |  |  |  |
