# Supplementary material for: Prevalence and genome features of lake sinai virus isolated from Apis mellifera in the Republic of Korea
Source: PLoS One. 2024 Mar 19;19(3):e0299558. doi: 10.1371/journal.pone.0299558 (PMC10950237; doi:10.1371/journal.pone.0299558)
Supplement: S3 Table — (DOCX) [file pone.0299558.s006.docx]

**S3 Table. Primers used for determining the Lake Sinai Virus genome.**

|  | **Primer name** | **Sequence 5′–3′** | **Position** | **Amplicon size (bp)** | **Note** |
| --- | --- | --- | --- | --- | --- |
| F1 | LSV2-1For | TGGTTACTGGTAATTGGCCGC | 76-96 | 1369 | Primers were designed based on NCBI accession number LR655824.1 |
| R1 | LSV2-1Rev | ACTGTCATAAAACTTATCATC | 1425-1445 |  |  |
| F2 | LSV2-2For | TKGCCGATTACTGTGCHGCYAC | 1384-1405 | 1184 |  |
| R2 | LSV2-2Rev | GGGTACCGCGATACCCATGAA | 2548-2568 |  |  |
| F3 | LSV2-3For | TGTGYTTATCACGATTTATGC | 2426-2446 | 1096 |  |
| R3 | LSV2-3Rev | GGAGCAACACGCYTAACRGCA | 3502-3522 |  |  |
| F4 | LSV2-4For | GTGTGCTGCGCCGATGGTCT | 3299-3318 | 1439 |  |
| R4 | LSV2-4Rev | AGGCATGTAAAATCCATCAGT | 4718-4738 |  |  |
| F5 | LSV2-5For | TCCGTCACTGTTAACAGTGA | 4562-4581 | 1349 |  |
| R5 | LSV2-5Rev | GCTGTCRCAACAACCAACAGC | 5891-5911 |  |  |
| F1 | LSV3-1For | CGGTCGTAYAACTTCCTGYCG | 28-48 | 1380 | Primers were designed based on NCBI accession number MZ821882.1 |
| R1 | LSV3-1Rev | CGAGCAGAACGGCAAAATGTG | 1387-1408 |  |  |
| F2 | LSV3-2For | GAAACGATTTGTCAGTTATTC | 1298-1318 | 1202 |  |
| R2 | LSV3-2Rev | CTCTGTCTAGGGCCACCAAAT | 2480-2500 |  |  |
| F3 | LSV3-3For | GCTCCGCCACCTCATCGTTCG | 2374-2394 | 1486 |  |
| R3 | LSV3-3Rev | CTGCGATTGCGTCGACGCCG | 3841-3860 |  |  |
| F4 | LSV3-4For | GTCTGTTCTTTACGCGTGAC | 3725-3745 | 1362 |  |
| R4 | LSV3-4Rev | GCATCCAAAGCCACCTGGTCG | 5067-5087 |  |  |
| F5 | LSV3-5For | CTGGTGTCGTGTGGATGGAGG | 4940-4960 | 1130 |  |
| R5 | LSV3-5Rev | GTCGCAACCACCAACACTGTT | 6050-6070 |  |  |
| F1 | LSV4-1For | TCATAACTTCCTAGCGTC | 1-20 | 1792 | Primers were designed based on NCBI accession number MZ821852.1 |
| R1 | LSV4-1Rev | GGCCTCAGCACGAAATCGCTC | 1773-1793 |  |  |
| F2 | LSV4-2For | TGGAACTGGGCDTCNATTCAC | 1677-1697 | 1310 |  |
| R2 | LSV4-2Rev | GTRATACCATACTCACTAAAKCC | 2967-2987 |  |  |
| F3 | LSV4-3For | GTCTATCCGCCCCTTATCCAT | 2903-2923 | 1694 |  |
| R3 | LSV4-3Rev | GTCTTRTCAGCATTGACCTC | 4578-4597 |  |  |
| F4 | LSV4-4For | GAGACTCAGGGATTTGTCAC | 4488-4507 | 1512 |  |
| R4 | LSV4-4Rev | CTTTAAGGAGGCTTACCAGCC | 5980-6000 |  |  |
