# Supplementary material for: Prevalence and genome features of lake sinai virus isolated from Apis mellifera in the Republic of Korea
Source: PLoS One. 2024 Mar 19;19(3):e0299558. doi: 10.1371/journal.pone.0299558 (PMC10950237; doi:10.1371/journal.pone.0299558)
Supplement: S4 Table — (DOCX) [file pone.0299558.s007.docx]

**S4 Table.** **Comparison of the near-complete genome feature of LSV2/Korea-2022 with reference strains in GenBank.**

| **NCBI accession No.** | **NS1** | | **RdRp** | | **CP** | | | **NS2** | | |
| --- | --- | --- | --- | --- | --- | --- | --- | --- | --- | --- |
|  | Length (nt) | nt identity (%) | Length (nt) | nt identity (%) | Length (nt) | nt identity (%) | Length (nt) | | nt identity (%) |  |
| MT732482.1 | 2538 | 98.6 | 1869 | 97.8 | 1563 | 97.3 | 453 | | 98.2 |  |
| MZ821886.1 | 2538 | 98.0 | 1869 | 97.5 | 1563 | 97.0 | 453 | | 98.0 |  |
| MZ821910.1 | 2538 | 97.6 | 1869 | 97.5 | 1563 | 98.2 | 453 | | 98.5 |  |
| MZ821899.1 | 2538 | 97.3 | 1869 | 97.4 | 1563 | 98.0 | 453 | | 98.5 |  |
| MZ821912.1 | 2538 | 97.2 | 1869 | 97.3 | 1563 | 98.1 | 453 | | 98.9 |  |
| MZ821869.1 | 2538 | 97.2 | 1869 | 97.3 | 1563 | 97.4 | 453 | | 98.7 |  |
| MZ821857.1 | 2538 | 96.7 | 1869 | 96.3 | 1563 | 95.7 | 453 | | 95.8 |  |
| MZ821894.1 | 2538 | 96.5 | 1869 | 96.5 | 1563 | 96.0 | 453 | | 98.2 |  |
| MZ821876.1 | 2538 | 96.5 | 1869 | 96.1 | 1563 | 95.7 | 453 | | 97.6 |  |
| MZ821872.1 | 2538 | 96.4 | 1869 | 96.6 | 1563 | 96.2 | 453 | | 97.4 |  |
| MZ821853.1 | 2547 | 96.4 | 1869 | 96.3 | 1563 | 96.0 | 453 | | 96.6 |  |
| OL803842.1 | 2538 | 94.5 | 1869 | 96.1 | 1563 | 93.4 | 453 | | 96.2 |  |
| OL803841.1 | 2538 | 94.5 | 1869 | 96.0 | 1563 | 93.4 | 453 | | 97.0 |  |
| OL803843.1 | 2538 | 94.5 | 1869 | 96.0 | 1563 | 93.2 | - | | - |  |
| KY465711.1 | 2538 | 93.6 | 1869 | 92.6 | 1563 | 94.8 | 453 | | 95.8 |  |
| KY465710.1 | 2538 | 93.6 | 1869 | 92.9 | 1563 | 94.6 | 453 | | 96.3 |  |
| KY465707.1 | 2547 | 93.6 | 1869 | 92.4 | 1563 | 93.3 | 453 | | 95.6 |  |
| NC_035467.1 | 2538 | 93.6 | 1869 | 92.9 | 1563 | 94.6 | 453 | | 96.3 |  |
| OL803840.1 | 2426 | 94.4 | 1869 | 94.1 | 1563 | 93.3 | - | | - |  |
| LR655824.1 | 2538 | 93.2 | 1869 | 92.4 | 1563 | 92.4 | 453 | | 96.5 |  |
| KY465709.1 | 2538 | 93.1 | 1869 | 92.1 | 1563 | 94.2 | 453 | | 95.4 |  |
| KY465713.1 | 2538 | 92.7 | 1869 | 91.9 | 1563 | 93.4 | 453 | | 95.4 |  |
| KY465708.1 | 2538 | 92.6 | 1869 | 91.9 | 1563 | 94.1 | 453 | | 96.5 |  |
| KY354241.1 | 2547 | 92.6 | 1983 | 92.2 | 1563 | 91.7 | 453 | | 94.7 |  |
| NC_035116.1 | 2547 | 92.6 | 1983 | 92.2 | 1563 | 91.7 | 453 | | 94.7 |  |
| KY465706.1 | 2547 | 92.3 | 1869 | 90.8 | 1566 | 92.5 | 453 | | 95.4 |  |
| HQ888865.2 | 2538 | 93.1 | 1869 | 93.5 | 1563 | 94.4 | 451 | | 96.9 |  |
| KY465712.1 | 2538 | 92.1 | 1869 | 91.1 | 1563 | 92.0 | 453 | | 94.5 |  |

The nucleotide lengths of NS1, RdRp, CP, and NS2 in LSV2/Korea-2022 were 2,538, 1,869, 1,563, and 453, respectively. “-” No information. nt: nucleotide; NS1: first nonstructural protein region; RdRp, RNA-dependent RNA polymerase; CP, capsid protein; NS2, second nonstructural protein region.
