# Supplementary material for: Prevalence and genome features of lake sinai virus isolated from Apis mellifera in the Republic of Korea
Source: PLoS One. 2024 Mar 19;19(3):e0299558. doi: 10.1371/journal.pone.0299558 (PMC10950237; doi:10.1371/journal.pone.0299558)
Supplement: S5 Table — (DOCX) [file pone.0299558.s008.docx]

**S5 Table. Comparison of the near-complete genome feature of LSV3/Korea-2022 with reference strains in GenBank.**

| **NCBI accession No.** | **NS1** | | **RdRp** | | **CP** | | **NS2** | |
| --- | --- | --- | --- | --- | --- | --- | --- | --- |
|  | Length (nt) | nt identity (%) | Length (nt) | nt identity (%) | Length (nt) | nt identity (%) | Length (nt) | nt identity (%) |
| Z821900.1 | 2589 | 98.7 | 2070 | 98.5 | 1557 | 97.6 | 477 | 98.5 |
| MZ821885.1 | 2589 | 98.6 | 2070 | 98.5 | 1557 | 97.0 | 477 | 98.5 |
| MZ821866.1 | 2589 | 98.5 | 2070 | 98.8 | 1557 | 97.6 | 477 | 98.7 |
| MZ821854.1 | 2589 | 98.4 | 2070 | 98.6 | 1557 | 97.7 | 477 | 98.7 |
| MZ821882.1 | 2589 | 98.4 | 2070 | 98.3 | 1557 | 97.6 | 477 | 98.5 |
| MZ821909.1 | 2589 | 98.1 | 2070 | 98.2 | 1557 | 97.5 | 477 | 98.5 |
| MZ821887.1 | 2589 | 97.9 | 2070 | 98.3 | 1557 | 97.9 | 477 | 98.7 |
| MZ821863.1 | 2589 | 97.9 | 2070 | 98.6 | 1557 | 97.9 | 477 | 98.1 |
| MZ821914.1 | 2589 | 97.8 | 2070 | 98.6 | 1557 | 97.8 | 477 | 97.7 |
| MZ821915.1 | 2589 | 94.6 | 2070 | 95.6 | 1557 | 94.0 | 477 | 96.4 |
| MZ821878.1 | 2589 | 94.4 | 2070 | 95.1 | 1557 | 93.6 | 477 | 96.9 |
| MZ821916.1 | 2589 | 86.0 | 2070 | 87.6 | 1557 | 82.0 | 477 | 89.3 |
| MH267700.1 | 2589 | 86.0 | 1869 | 87.3 | 1548 | 83.2 | 453 | 89.4 |
| MZ821907.1 | 2589 | 85.8 | 2070 | 87.5 | 1557 | 81.7 | 477 | 90.3 |
| MT636353.1 | 2526 | 85.3 | 2070 | 85.7 | 1557 | 84.3 | 453 | 90.2 |
| MH267699.1 | 2526 | 85.2 | 1845 | 85.8 | 1557 | 83.0 | 453 | 88.2 |
| OL803835.1 | 2589 | 85.0 | 1869 | 87.1 | 1557 | 84.6 | 453 | 89.1 |
| MZ821892.1 | 2589 | 84.8 | 2097 | 86.2 | 1557 | 82.5 | 477 | 88.2 |
| MZ821917.1 | 2589 | 84.6 | 2097 | 86.1 | 1557 | 82.0 | 477 | 88.2 |
| MZ821903.1 | 2589 | 84.6 | 2097 | 86.3 | 1557 | 82.2 | 477 | 88.2 |
| KY465720.1 | 1257 | 88.5 | 1665 | 87.4 | - | - | - | - |
| KY465719.1 | - | - | 1152 | 85.0 | 1644 | 83.9 | 453 | 89.7 |

The nucleotide lengths of NS1, RdRp, CP, and NS2 in LSV3/Korea-2022 were 2,589, 1,869, 1,557, and 453, respectively. “-” No information. nt: nucleotide; NS1: first nonstructural protein region; RdRp, RNA-dependent RNA polymerase; CP, capsid protein; NS2, second nonstructural protein region.
