# Supplementary material for: Prevalence and genome features of lake sinai virus isolated from Apis mellifera in the Republic of Korea
Source: PLoS One. 2024 Mar 19;19(3):e0299558. doi: 10.1371/journal.pone.0299558 (PMC10950237; doi:10.1371/journal.pone.0299558)
Supplement: S6 Table — (DOCX) [file pone.0299558.s009.docx]

**S6 Table. Comparison of the near-complete genome feature of LSV4/Korea-2022 with reference strains in GenBank.**

| **NCBI accession No.** | **NS1** | | **RdRp** | | **CP** | | **NS2** | |
| --- | --- | --- | --- | --- | --- | --- | --- | --- |
|  | Length (nt) | nt identity (%) | Length (nt) | nt identity (%) | Length (nt) | nt identity (%) | Length (nt) | nt identity (%) |
| MZ821856.1 | 2598 | 97.7 | 1941 | 98.1 | 1557 | 97.9 | 453 | 98.0 |
| MZ821861.1 | 2598 | 97.5 | 1941 | 96.0 | 1557 | 97.5 | 453 | 98.2 |
| KX883223.1 | 2550 | 96.9 | 1869 | 96.8 | 1557 | 96.5 | 453 | 97.4 |
| MZ821852.1 | 2550 | 96.6 | 1941 | 96.8 | 1557 | 95.6 | 453 | 95.6 |
| MZ821850.1 | 2598 | 96.5 | 1941 | 96.5 | 1557 | 95.6 | 453 | 96.7 |
| MZ821860.1 | 2598 | 96.5 | 1941 | 96.4 | 1557 | 96.1 | 453 | 96.5 |
| MZ821864.1 | 2598 | 96.4 | 1941 | 96.3 | 1557 | 96.1 | 453 | 97.1 |
| MZ821913.1 | 2598 | 96.4 | 1956 | 96.6 | 1557 | 96.0 | 453 | 96.3 |
| MZ821893.1 | 2598 | 96.3 | 1941 | 96.1 | 1557 | 95.9 | 453 | 96.7 |
| MZ821859.1 | 2598 | 96.2 | 1941 | 96.0 | 1557 | 95.7 | 453 | 95.4 |
| MZ821901.1 | 2598 | 96.1 | 1941 | 96.1 | 1557 | 95.4 | 453 | 96.5 |
| MZ821911.1 | 2598 | 96.0 | 1941 | 96.2 | 1557 | 95.5 | 453 | 95.4 |
| MZ821905.1 | 2598 | 96.0 | 1941 | 95.9 | 1557 | 95.5 | 453 | 94.5 |
| MZ821867.1 | 2598 | 96.0 | 1941 | 95.8 | 1557 | 95.5 | 453 | 95.1 |
| MZ821855.1 | 2598 | 95.8 | 1941 | 96.0 | 1557 | 95.5 | 453 | 96.2 |
| MZ821884.1 | 2598 | 95.7 | 1941 | 95.6 | 1557 | 95.6 | 453 | 96.0 |
| MZ821871.1 | 2598 | 95.6 | 1941 | 95.7 | 1557 | 96.0 | 453 | 96.0 |
| MZ821875.1 | 2598 | 95.1 | 1941 | 95.5 | 1557 | 95.3 | 453 | 94.0 |
| MZ821862.1 | 2598 | 94.9 | 1941 | 95.8 | 1557 | 95.1 | 453 | 95.8 |
| MZ821918.1 | 2598 | 94.8 | 1941 | 95.4 | 1557 | 95.0 | 453 | 94.9 |
| OL803850.1 | 2598 | 91.3 | 1941 | 91.7 | 1557 | 90.3 | 453 | 90.3 |
| KM886903.1 | 2598 | 90.4 | 1941 | 91.5 | 1557 | 90.6 | - | - |

The nucleotide lengths of NS1, RdRp, CP, and NS2 in LSV4/Korea-2022 were 2,550, 1,869, 1,557, and 453, respectively. “-” No information. nt: nucleotide; NS1: first nonstructural protein region; RdRp, RNA-dependent RNA polymerase; CP, capsid protein; NS2, second nonstructural protein region.
