# Supplementary material for: Prevalence and genome features of lake sinai virus isolated from Apis mellifera in the Republic of Korea
Source: PLoS One. 2024 Mar 19;19(3):e0299558. doi: 10.1371/journal.pone.0299558 (PMC10950237; doi:10.1371/journal.pone.0299558)
Supplement: S7 Table — (DOCX) [file pone.0299558.s010.docx]

**S7 Table. Detection of Lake Sinai Virus in *Varroa destructor* and *Apis mellifera*.**

| **Colony No.** | ***Varroa destructor*** | | | ***Apis mellifera*** | | |
| --- | --- | --- | --- | --- | --- | --- |
|  | **LSV2** | **LSV3** | **LSV4** | **LSV2** | **LSV3** | **LSV4** |
| 1 | - | 31.1 ± 0.8^*^ | - | - | 31 ± 1.3 | 33.1 ± 0.8 |
| 2 | - | - | - | - | - | 25.4 ± 2.2 |
| 3 | 30.2 ± 3.6^*^ | 32 ±1.5 | - | 19.5 ± 2.1 | 30.4 ± 2.3 | 31.4 ± 4.6 |
| 4 | - | 31.1 ± 0.6 | - | - | 27.2 ± 3 | - |
| 5 | - | - | - | - | 34.19 ± 1.6 | - |
| 6 | - | - | - | 26 ± 0.3 | - | - |
| 7 | - | 31.9 ± 0.6 | - | - | 26.7 ± 5 | - |
| 8 | - | - | - | - | - | 33.5 ± 2.5 |
| 9 | - | 31.9 ± 1.5 | - | - | 30.4 ± 6.3 | - |
| 10 | - | - | - | - | 26.4 ± 5.9 | - |
| 11 | - | 32.7 ± 1.3 | - | 32.8 ± 0.4 | 21 ± 1.8 | - |
| 12 | 31.8 ± 2.9 | - | - | 29.2 ± 3.6 | - | 32.9 ± 2 |

^“*”^ Values are the data of cycle thresholds (*C_t_*) ± SD. A *C_t_* value of ≤ 35 was considered positive. “-” No detection. LSV2: Lake Sinai virus 2; LSV3: Lake Sinai virus 3; LSV4: Lake Sinai virus 4.
