# Supplementary material for: Prevalence and genome features of lake sinai virus isolated from Apis mellifera in the Republic of Korea
Source: PLoS One. 2024 Mar 19;19(3):e0299558. doi: 10.1371/journal.pone.0299558 (PMC10950237; doi:10.1371/journal.pone.0299558)
Supplement: Attachment — Submitted filename: Response to Reviewers.docx [file pone.0299558.s012.docx]

**Responses to Reviewers’ Comments**

*We appreciate the reviewers for their invaluable comments. As explained below, we have revised our original manuscript and materials in response to all of the reviewers’ comments. What follows are our point-by-point responses to the comments.*

**Reviewer #1**: Summary: I appreciate the authors’ efforts to clarify their methodology and add context to their Introduction and Discussion. I still feel that the potential for primer bias is not fully acknowledged, despite the clear potential evident in Supplemental Figure S1, for example. The authors may have felt that the LSV2 accession used for primer design well represented previous results in the ROK, and they could also cite Hou et al. 2023 https://doi.org/10.1111/mec.16987, which shows continental genetic clustering within all the major LSV clades, as justification for their approach. Nonetheless, the paper makes many statements that gloss over ascertainment issues, such as in the abstract: “This study aimed to assess the prevalence of ... the LSVs ...from the ROK”). These claims overlook the fact that LSV1 is not assayed and assays for LSV2/LSV3/LSV4 have known primer mismatches with LSV2, LSV3, or LSV4 clade members. See also lines 81-86, line 109, and line 251 as examples where LSV is used instead of the specific LSV genotypes for which an assay was developed.

Responses:

We acknowledge the valuable feedback and insights provided by Reviewer 1. This study investigated the presence of previously unreported honeybee pathogens associated with Colony Collapse Disorder (CCD) or Severe Winter Loss (SWL) in the Republic of Korea. Samples were collected in 2022 and analyzed for Lake Sinai Viruses 1 (LSV1) and 2 (LSV2), known CCD/SWL agents. Initially, primers for both viruses were employed as described in Runckel *et al.* (2011) (<https://doi.org/10.1371/journal.pone.0020656>). Although PCR amplification suggested LSV1 detection based on target size of PCR product. Sequence analysis surprisingly revealed a high similarity (97.83%) to the reference LSV4 strain (NCBI accession no: MZ821852.1). On the other hand, the similarity to the LSV1 strain was lower, at only around 90.86% (NCBI accession no: KY65700.1). This finding suggests that the isolated virus is LSV4, despite initial assumption based on product size (Shown in Figure 1). In our study, although the LSV2-specific primers employed by Runckel et al. (2011) were not utilized due to unsuccessful application in our samples. The detection of LSV4 based on primers designed for LSV1 and LSV2 raises the possibility of recombinant strains emerging from co-infection events.

**Figure 1.** **Alignment of Lake Sinai Virus 1 and 4 sequences.** The sequences of LSV4 from honeybee samples were sequenced using forward and reverse primers of LSV1. The reference sequence of LSV1 (with NCBI accession nos: KY465704.1, KY465701.1, and LR596015.1) and LSV4 (with NCBI accession nos: MZ821852.1 and KX883223.1).

While the initial focus of our study did not include LSV1, however, we will add some information addressing the investigation of LSV1 in the discussion section, provides additional insights and highlights the complex nature of LSV diversity and interrelationships. Our results may be partially influenced by the inherent limitations of the PCR technique, specifically primer bias. The imperfect primer-target sequence pairing can lead to underrepresentation or overestimation of specific variants. This challenge is particularly amplified by the high degree of sequence variability within LSV strains, further emphasizing the need for optimized detection methods. Given the observed prevalence of LSVs and their high degree of geographic variation, we strongly advocate for deeper investigation into this viral complex within South Korean honeybee colonies. Such more in-depth research on this virus strain in honeybee populations, employing optimized detection methods and comprehensive analyses, will enhance our understanding of LSVs diversity and its potential impact on bee health.

**Some additional comments are provided below.**

The authors continue to state that they have generated “complete genome sequences of LSV” using primers that do not start at position 1 of any reference and do not end at the final position of any reference. It is a minor point of no import for any conclusions, but these overlapped sequences cannot be called “complete” genomes, even if 99% of the bases are captured by the method. Line 221, for example, would need to be changed to “near-complete genome” or “partial genome”. Similarly, in the abstract the phrase “the first genome feature of the Korean “should be changed to “the first near-complete genome sequences of Korean”. Nor is it certain whether the overlapped amplicons all come from a single virus strain, ie the assemblies could be chimeric. It has been previously documented that single individuals can harbor diverse strains (e.g. Ravoet et al; Iwanowicz et al.).

Response: We thank the reviewer for their positive comments. Modified as suggested.

Lines 53-54: Not correct as written, change to “with distinctive features that may be absent in some LSV clades”? Also, does “the evolutionary process” mean “replication” (line 54)?

Responses: This has been corrected (lines 53-58)

Line 65-66: “genotypes” rather than “gene types”

Responses: Modified as suggested.

The authors continue to state “LSV may cause behavioral changes in honeybees, leading to worker bees abandoning their hives” (lines 77-78). The cited references only show that LSV was found to be more prevalent in weak/collapsed hives than healthy. Nothing can be said about “behavioral changes in honeybees” caused by LSV. The fact that there may be a behavorial component to the colony collapse phenomenon does not mean LSV causes any behavior.

Responses: This has been corrected in lines 80-84

Lines 142-143: I think “The alignment was generated and trimmed to the RdRp gene region in Bioedit” would be clearer.

Responses: Modified as suggested.

Lines 152-153: The BLASTN software is different from the database it was used to search. The default for BLASTN for the NCBI web service is the nt database, but any database can be used in principle. If nt was used, please explicitly state so for completeness/repeatability.

Responses: This has been corrected in lines 147-149

Line 162: As the ANOVA is performed on proportions of positives within colonies, have the authors verified that the data are reasonably distributed? If not, a Kruskal-Wallis nonparametric test could be used instead.

Responses: Thank you for your valuable suggestion regarding the potential non-normality of our data for analyzing LSV variants across geographically distinct samples. We fully appreciate the importance of data distribution assumptions for statistical tests and agree that the Kruskal-Wallis test, as you recommended, will be a more appropriate option in this case. We will implement this change to ensure our analysis accurately reflects the nature of our data and provides reliable conclusions.

Line 166: “detection rate” instead of “infection rate”

Responses: Modified as suggested.

Comment: Line 168-170: if the overall detection rate of LSV2 was greater than 60%, how can the values on line 170 be considered high?

Responses: We have included this information in further detail in lines 176-180.

“LSV2 was detected in 266 honeybee colonies was 60.9% (162/266) in ROK. A comparison of the presence of LSV2 in different provinces of ROK revealed a statistically significant variation in LSV2 infection rates (*p* = 0.011). Among the LSV2-positive samples, the highest prevalence of LSV2 was observed in Gyeongsangbuk-do (32.5%), followed by Gyeongsangnam-do (21.6%), and Jeollanam-do (17.3%) (Fig 2).”

Lines 176-178: the %id is lower for the LSV2 accession than the LSV3 and LSV4 accessions, in reverse order to what the text states.

Responses: Sequence analysis revealed that the identified sequences showed the greatest resemblance to LSV2 (94.5%; MZ281853), followed by LSV3 (97.6%; MZ821878) and LSV4 (98.4%; MZ281893). This lower percent of identified sequences for LSV2 compared to the others is likely due to several nucleotide mutations within the short-amplified region (218 bp) compared to the reference sequence in NCBI (as shown in Figure 2)

**Figure 2.** **Alignment of the nucleotide sequences of Lake Sinai Virus 2.** The sequences of LSV2 from honeybee samples were sequenced using forward and reverse primers of LSV2. The reference sequence of LSV2 was compared in BioEdit program (with NCBI accession nos: HQ88865.2 and LR655824.1)

Line 182: this could just be due to primer bias? European like lineages might be present in ROK apiaries but not amplified.

Responses: We appreciate the reviewer's valuable feedback regarding potential primer bias and its impact on the observed clustering of LSV isolates in our study. We fully acknowledge this concern and have taken several steps to minimize its influence. We designed specific primer pairs for each LSV variant (LSV1, LSV3, and LSV4) to enhance amplification specificity and reduce potential bias. The target strains were successfully amplified with their corresponding primers, demonstrating the functionality of our approach in detecting known LSV variants in the ROK. While not perfect, our primers have shown potential in amplifying diverse LSV strains circulating in ROK. The unexpected detection of LSV3 and LSV4 using primers designed for LSV2, and LSV4 using primers designed for LSV1/LSV2, indeed highlights the remarkable genetic diversity within Korean LSV isolates. This underscores the need for further investigation employing wider genomic regions beyond our current approach would provide a more comprehensive understanding of LSV diversity.

We agree that further studies are necessary to explore the full spectrum of LSV variants in ROK. Expanding our research to encompass a wider range of South Korean honeybee colonies is crucial to detect potential LSV variants related to European lineages or other sources. By incorporating these suggestions, we hope to refine our study and contribute to a more comprehensive understanding of LSV diversity and its potential impact in the region.

“Line 274: In the present study, we also detected LSV in *V. destructor* mites, as these sources have been proposed as potential transmission routes for this emerging virus within Korean honeybee apiaries” What was the nature of the varroa samples? Were they collected from sticky boards, sugar roll, directly from parasitized adults or pupae? Were individual mites analyzed or pools? How were the twelve colonies selected for this assay?

Responses: This study investigated the potential presence of LSVs in *V. destructor* mites associated with honeybee colonies. Mites were collected using the sugar-shake method described by Dietemann et al. (2012) (<https://doi.org/10.3896/IBRA.1.52.1.09>). Briefly, the *Varroa* mites were collected using one spoon of powdered sugar poured on over 300 honeybees kept in a jar with a mesh. The five mites were pooled from each colony for total nucleic acid extraction.

To assess LSVs prevalence in mites, twelve honeybee colonies confirmed positive for LSVs in bees (2022 samples) were revisited in 2023. Randomly selected *Varroa* mites were collected from these confirmed-positive colonies and tested for LSVs presence. While this approach provides valuable insights, we acknowledge that it might not capture the full spectrum of LSVs prevalence across the entire range of colony health statuses. Therefore, in this study, we have confined this information to the discussion section.

Also, what is your conclusion regarding the fact that your LSV4 assay was never positive in mites, although numerous positives in honeybees were obtained? Are patterns of discordance between the two species in S7 Table a matter of chance sampling or do you believe some species-level difference in LSV host interaction is occurring? Some discussion would be appreciated.

Responses: We appreciate the reviewer’s thoughtful evaluation. We have added more information in the discussion (lines 300-307).

“This study showed the presence of LSV2 and LSV3 in *V. destructor* mites associated with honeybee colonies but failed to detect LSV4 in any mite samples across the twelve surveyed honeybee colonies (S7 Table). This could be because LSV4 exists at very low concentrations within mites, making detection more challenging than for the identified LSV2 and LSV3 strains. Alternatively, the LSV4 might exhibit uneven distribution within bee colonies, leading to an inconsistent presence in mite populations. Additionally, the chosen sampling methodology might not have been sufficiently sensitive to detect LSV4, requiring the exploration of alternative approaches.”

Line 288-290 should note this is consistent with results of Hou et al. 2023, but also potentially an artifact of primer bias.

Responses: This has been added at lines 285-286 and line 325
